# Detection and Multigene Typing of ‘*Candidatus* Phytoplasma solani’-Related Strains Infecting Tomato and Potato Plants in Different Regions of Turkey

**DOI:** 10.3390/pathogens11091031

**Published:** 2022-09-11

**Authors:** Behçet Kemal Çağlar, Eray Şimşek

**Affiliations:** 1 Department of Plant Protection, Faculty of Agriculture, Çukurova University, Adana 01330, Turkey; 2 Department of Plant Protection, Faculty of Agriculture, Harran University, Şanlıurfa 63290, Turkey

**Keywords:** ‘*Candidatus* Phytoplasma solani’, potato, tomato, *Sec*Y, *Vmp*1, *Tuf*, *16SrDNA*

## Abstract

‘*Candidatus* Phytoplasma solani’ (‘*Ca*. P. solani’) is a crop pathogen that is a member of the 16SrXII-A ribosomal subgroup. It is also known as stolbur phytoplasma and causes yield losses in several important crops, especially in Solanaceous crops. Different strains of the pathogen are regularly reported all over the world, particularly in the Mediterranean region. In this study, the determination of genetic diversity for the pathogen infecting tomatoes and potatoes was carried out by using multilocus sequence typing analysis for the *Tuf*, *Sec*Y, and *Vmp*1 genes to gain insight into the epidemiology of ‘*Ca*. P. solani’ in Turkey. Genetic diversity of the phytoplasmas was investigated by sequence-based phylogenetic analyses and in silico RFLP analysis of related genes. It was determined that all ‘*Ca*. P. solani’-related strains infecting tomatoes and potatoes were tuf-b, which is linked to field bindweed (*Convolvulus arvensis* L.). Tomato or potato-infecting ‘*Ca*. P. solani’-related strains showed similarities with each other; however, the isolates collected from different plants showed genetic differences in terms of the *Sec*Y gene. This study indicates that the highest genetic variability of collected samples was found in the *Vmp*1 gene. *Rsa*I-RFLP analysis of TYPH10F/R amplicons showed that potato-infecting ‘*Ca*. P. solani’-related strains were found to be similar to some existing V types. However, the V-type of tomato-infecting isolates is not similar to any previously reported V-type. The results indicate that there could be an important genetic diversity of ‘*Ca*. P. solani’-related phytoplasmas in Turkey. This could indicate various ways in which the pathogen has adapted to the two host plants as a consequence of the various *Vmp*1 gene rearrangements seen in these two plant hosts. Obtained results also indicate that the epidemiology of ‘*Ca*. P. solani’-related phytoplasmas in the tomato and potato agroecosystem may be better understood with the use of molecular data on the complex of vmp-types.

## 1. Introduction

Potato (*Solanum tuberosum* L.) and tomato (*Solanum lycopersicum* L.) are two significant crops of the Solanaceae family, with an annual production of 13.095.258 and 5.100.674 tons, respectively, in Turkey [1]. As with many other wild and cultivated plants, tomato and potato plants are seriously affected by phytoplasma agents, which are phytopathogenic Mollicutes invading phloem tissues (the sieve elements) of the host plant. The diseases caused by phytoplasmas have been causing significant economic losses. The most common phytoplasma agent detected in tomato and potato production areas in Turkey is ‘*Ca*. phytoplasma solani’ [2,3,4,5]. ‘*Ca*. phytoplasma solani’ was also detected and characterized in different plant species, e.g., sesame [2,3,4,5], grapes [6], maize [7], pomegranate [8], and cucumber [9] in Turkey. ‘*Ca*. P. solani’ causes “bois noir” in vineyards and leaf redness diseases in maize plants [10]. Bois noir is one of the serious phytoplasma diseases that affect grapevines and is linked to stolbur phytoplasma [11]. ‘*Ca*. P. solani’ also results in stolbur (yellow type disease) in potato, tomato, pepper, tobacco, carrot, celery, garden bean, parsley, etc. [11]. Stolbur disease is characterized by a significant yield loss. The polyphagous insect vector *Hyalesthes obsoletus* (Cixiidae), which can complete its life cycle on just a few plant species, typically transmit the pathogenic agent ‘*Ca*. P. solani’ from plant to plant [12,13]. Because of its intricate ecological and epidemiological complex life cycle, it can adapt to different agroecosystems. Therefore, spreading the disease agent via insects or other crop plants into various geographical regions is inevitable [14,15]. A symptomless period in crop plants for a certain amount of time may also mask the disease is being detected unless delicate molecular methods have been employed. Therefore, rapid and simple phytoplasma detection/typing methods have been in great demand [16]. The use of the *16SrDNA* sequence often does not provide enough information to carry out most of the epidemiological investigations based on strain discrimination [17]. Multigene genotyping techniques have been developed to distinguish different phytoplasma strains. ‘*Ca*. Phytoplasma solani’ has many different strains that have been molecularly characterized based on the *Tuf*, *Sec*Y, *Vmp*1, and *Stamp* sequencing. Many researchers have developed molecular typing methods, including several genes [11,18,19]. Molecular detailed characterization or typing of ‘*Ca*. P. solani’ has so far identified 33, 59, and 80 genetic variants based on the sequences of *Sec*Y, *Stamp*, and *Vmp*1, respectively [14,20]. Additionally, the *Tuf* gene sequence studies indicated the existence of three ‘*Ca*. P. solani’ Tuf-types (a, b1, and b2) are linked to various ecological systems [14,21,22,23]. The housekeeping gene *Tuf* encoding the elongation factor Tu was examined to ascertain the molecular epidemiology of ‘*Ca*. P. solani’ [24]. RFLP characterization of *Tuf* revealed two main types: *Tuf* a, which infects stinging nettle (*Urtica dioica*), and *Tuf*-b (later called *Tuf*-b1), which is linked to field bindweed (*Convolvulus arvensis*) [25]. In a limited area of Germany, a third minor genotype known as *Tuf*-c was also discovered in hedge bindweed (*Calystegia sepium*) as an alternate host plant [24]. By the sequence analysis, novel tuf-type variants were associated with stinging nettle, designated as *Tuf*-b2 [26,27] and *Tuf*-ab [28,29] in vineyards in Austria, Macedonia, Croatia, and Montenegro.

*Sec*Y is an integral membrane protein that forms a heterotrimeric channel-like structure with *SecE* and *SecG* in the *Sec*Y secretion pathway of preproteins via the bacterial cell membrane, which is mediated by ATPase [30,31]. Because of its capacity to interact with numerous targeting factors, chaperones, and accessory proteins, the *SecYEG* translocon is globally conserved and plays a vital role in bacterial protein transport regulating protein translocation across the cytoplasmic membrane [32] because protein translocation is a ubiquitous and necessary mechanism that permits secretory proteins to leave cells and membrane proteins to integrate into lipid bilayers [33]. 

*Vmp*1 and *Stamp* are the other housekeeping genes encoding membrane proteins. Phylogenetic analyses of *Vmp*1 and *Stamp*-based molecular markers allow us to better understand how phytoplasma population structures and dynamics are formed [34,35]. The *Vmp*1 gene encodes a 557-amino-acid protein with a suspected signal peptide and a possible C-terminal transmembrane domain [36]. The mature 57.8 kDa Vmp1 protein is presumably embedded in the phytoplasma membrane, with a significant hydrophilic portion exposed to the cell surface [36].

Understanding the genetic diversity of phytoplasma is a powerful tool for analyzing their epidemiology and putting proper disease monitoring and management in place. The primary focus of this study was to investigate the genetic diversity of ‘*Ca*. P. solani’ infecting tomato and potato plants to highlight the potential of genetic variants in the country and what they are. The presence of ‘*Candidatus* Phytoplasma solani’-related strains have been previously reported in Turkey. However, no studies have been conducted yet in terms of studies on genes that provide epidemiological information. Based on the nested-PCR assay, we first identified the causal agents of the symptomatic tomato and potato sampled from the Kahramanmaraş and Adana regions. Afterward, some informative genes were used for detailed characterization of the pathogen to evaluate the epidemiologically informative *Tuf*, *Vmp*1, and *Sec*Y genes of ‘*Ca*. P. solani’-related strains in naturally infected tomato and potato to elucidate genetic relationships that could have importance in disease transmission in the region. Our data also prove that different ‘*Ca*. P. solani’ lineages infect tomato and potato plants in close regions. 

## 2. Results

### 2.1. Phytoplasma Detection and Symptom Observation 

**Figure 1:**
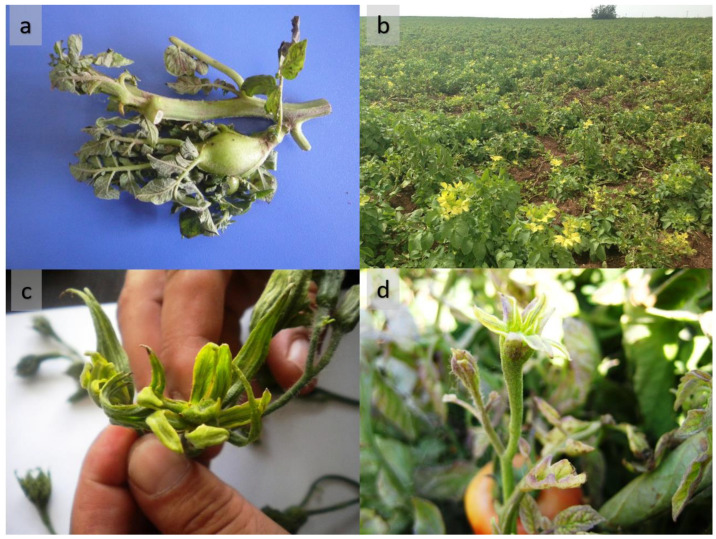
Disease symptoms related to the presence of ‘*Ca*. P. solani’-related strains in tomato and potato. (**a**) symptoms of potato stolbur phytoplasma of aerial tubers, (**b**) stolbur-infected potato plants in the field have yellowing and upward rolling top leaves, (**c**,**d**); Stolbur phytoplasma-infected tomato plants exhibited signs of sepal hypertrophy.

Potato plants with leaf yellowing or reddish discoloration with rolling leaf symptoms and tomato plants with floral abnormalities with sepal hypertrophy symptoms were observed and sampled from the surveyed fields (Figure 1). The presence of plants with these symptoms at the edge of the field raised suspicion of vector-borne disease infection. Guided sampling was performed to collect diseased plants, so all samples were taken from symptomatic plants. P1/Tint and F2n/R2 primer pairs were used to amplify DNA bands (about 1.2 kb) from 16 symptomatic leaf tissues and the positive control (data not shown). However, no bands were observed from the asymptomatic plants and negative control. 

**Figure 2:**
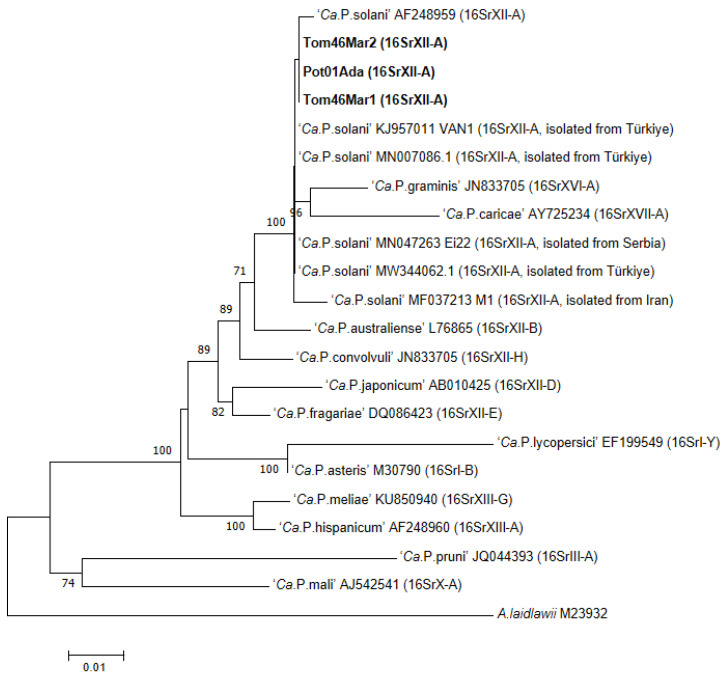
The Phylogenetic tree was constructed using ~1.2 kbp 16SrDNA gene fragments from the study’s phytoplasmas (bold) and the other phytoplasma species obtained from GenBank. The ‘*Ca*. P. solani’ strains detected in Turkey’s border neighbors are also included in the tree. MEGA7-created a neighbor-joining analysis was performed. At the nodes of the phylogenetic tree, bootstrap values greater than 70% were provided after 1000 bootstrap repeats. A distance of 0.01 substitutions per nucleotide site is represented by the scale bar. GenBank accession numbers and ribosomal subgroups are reported on the right of the strain names. *A. laidlawii* was employed as the outgroup to root the tree.

The representative sequences were chosen and submitted to GenBank under the accession numbers ON856337, ON856338, and ON856338, respectively. The 16SrDNA partial sequences of positive samples showed 100% identity with each other and 99.70% with that of ‘*Ca*. P. solani’ (GenBank accession number AF248959). Phylogenetic analysis based on 16SrDNA was also performed to classify the subgroup of phytoplasmas. According to the phylogenetic analysis results, all 16 positive samples were found to be infected with 16SrXII-A group phytoplasmas (Figure 2). While constructing the phylogenetic tree, one representative isolate was selected from each type based on other genes.

### 2.2. Sequence Typing by RFLP Analyses and Phytogenetic Relationships

**Figure 3:**
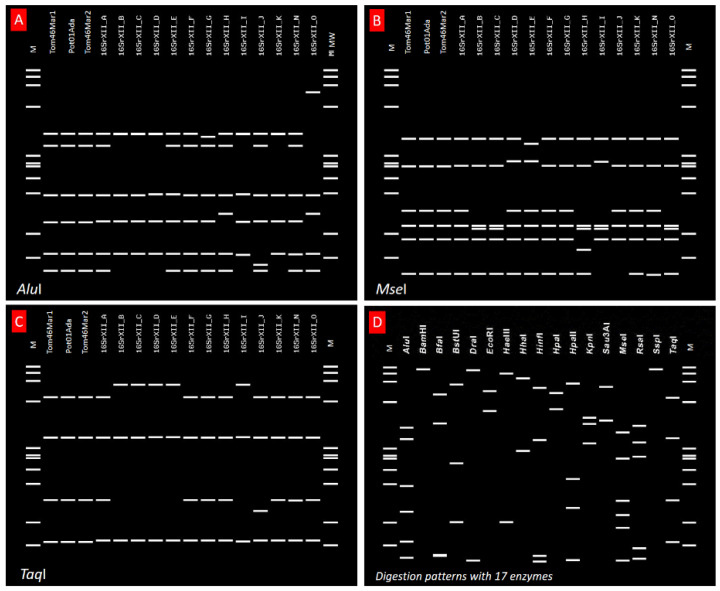
In silico RFLP patterns discriminating ‘*Ca*. P. solani’-related genotypes obtained from tomatoes and potatoes using *i*Phyclassifier. (**A**–**C**) are *Alu*I, *Mse*I, and *Taq*I digestions, respectively, indicating that the isolates are in subgroup 16SrXII-A. (**D**): the digestion profile of all samples with RFLP enzymes commonly used in phytoplasma 16Sr grouping. M: ϕX174 DNA-HaeIII Digest ladder (size range: 72, 118, 194, 234, 271, 281, 310, 603, 872, 1078, 1353 bp).

The first identification based on the 16S rDNA was accomplished using iPhyClassifier based on in silico RFLP patterns (similarity coefficient 1.00), which showed that the discovered phytoplasma belonged to the 16SrXII-A subgroup. Distinctive enzyme digestion patterns such as AluI, MseI, TaqI, and 14 others are shown in Figure 3. Based on the 16SrDNA gene, all isolates were found to exhibit the same RFLP profile. Since additional genes were recommended as criteria for thorough differentiation of ‘*Ca*. Phytoplasma’ species [37], classification was performed using sequence typing for three epidemiologically informative genes, *Tuf*, *Vmp*1, and *Sec*Y.

**Figure 4:**
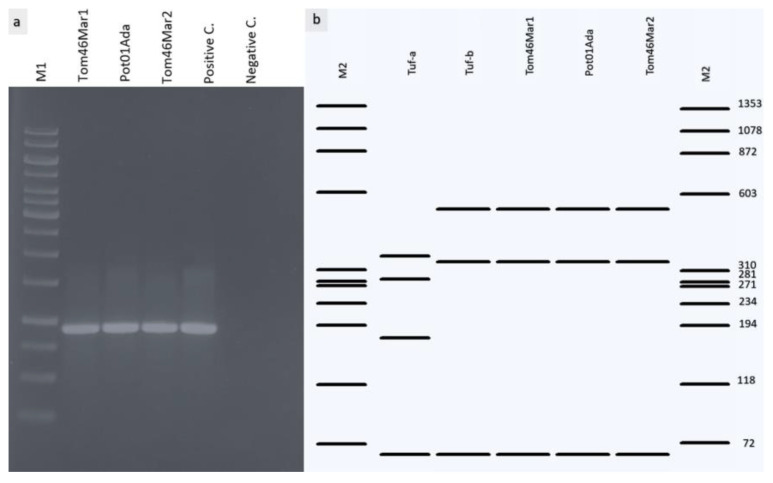
Wet and in silico gel images related to Tuf amplicons. (**a**) 1% agarose gel image of TufAYf/r amplicons. (**b**) RFLP analysis of the fTufAY/rTufAY amplicons of the elongation factor Tu (*Tuf*) gene with *Hpa*II. Only ‘*Candidatus* Phytoplasma solani’ *Tuf*-type b was detected. M1: 1 kb DNA ladder and M2: ϕX174 DNA-HaeIII Digest ladder.

*Hpa*II and *Rsa*I restriction enzymes performed RFLP studies on the *Tuf* and *Vmp*1 gene amplicons, respectively. All 16 positive plant samples yielded successful amplification of the Tuf gene with Tuf1f/r, and TufAYf/r primers gave the expected ~0.96 kbp PCR product (Figure 4a). The HpaII restriction profiles showed the presence of the bindweed-associated Tuf-b type as defined by [38] in all infected tomato and potato samples (Figure 4b). The Tuf gene partial sequences of positive samples showed 100% identity with each other and 99.89% with that of ‘Ca. P. solani’ (GenBank accession number JQ797670.1) and representative sequences were selected and submitted to GenBank under the accession numbers ON864440, ON864441, and ON864442, respectively.

**Figure 5:**
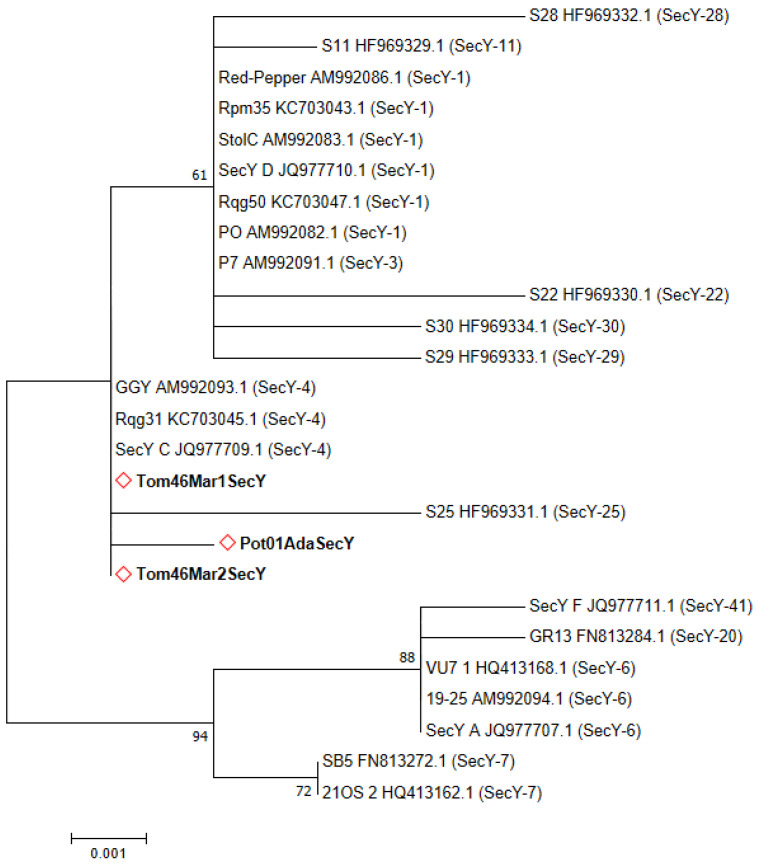
Unrooted phylogenetic tree constructed using ~0.9 kbp *Sec*Y gene fragments from ‘*Ca*. P. solani’ strains representing *Sec*Y sequence variants. The evolutionary history was inferred by using the Maximum Likelihood method based on the Tamura-Nei model. The neighbor-joining approach was used to do the minimum evolution analysis, and the bootstrap was reproduced 1000 times. The names of the strains and their GenBank accession numbers are shown in the figure, and the nucleotide sequences acquired in this study are highlighted with red diamonds. *Sec*Y types are shown in parentheses.

*Sec*Y gene sequences were determined for 16 samples from Adana and Kahramanmaraş. All samples gave the expected ~0.9 kbp bp PCR amplicons with PosecF1/R1 and PosecF3/R3 primer pairs (data not shown). Regardless of the region from which it is isolated, all ‘Ca. P. solani’ isolates infecting tomatoes were found identical to each other in terms of partial *Sec*Y gene fragments. Potato-infecting ‘*Ca*. P. solani’ isolates were found to be identical too; however, when the *Sec*Y gene fragments infecting tomato and potato plants were compared, it was detected that 99.88% similarity according to the blast analysis. Representative sequences were selected and submitted to GenBank under the accession numbers ON885880, ON885881, and ON885882, respectively. Phylogenetic analysis of ‘*Ca*. P. solani’-related strains infecting tomato and potato based on the *Sec*Y gene showed clustering of all strains in a close cluster to SecY-4-type phytoplasmas (Figure 5). 

**Figure 6:**
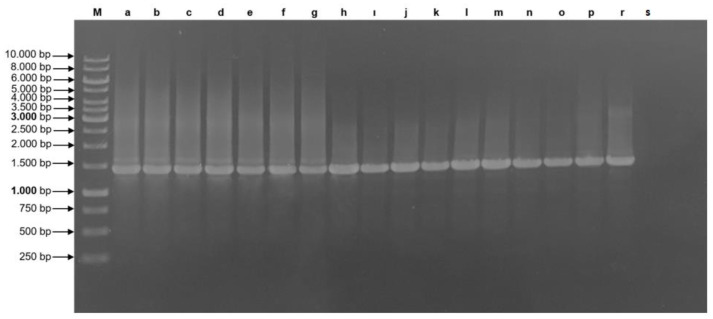
TYPH10F/R PCR products based on *Vmp*1 gene on 1% agarose gel., a–p: tomato and potato samples from Turkey. r and s: positive and negative control, respectively. M: 1 kb DNA ladder.

**Figure 7:**
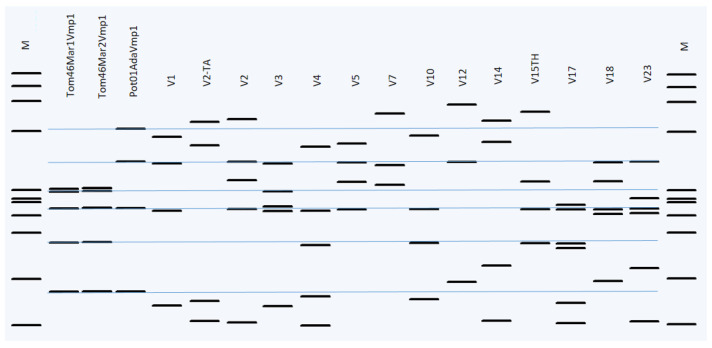
pDRAW32 1.1.147 generated *Rsa*I digestion RFLP patterns of TYPH10F/TYPH10R PCR fragments of the *Vmp*1 gene after virtual digestion with the reference strains. The *Rsa*I-RFLP profiles are labeled on top with the V nomenclature as suggested by the SEE-ERA.NET nomenclature [39]. The blue line has been added to make it easier to compare digested bands. M: marker HaeIII digested φX174 is shown on the right and left. Marker fragments length from top to bottom are 1353, 1078, 872, 603, 310, 281, 271, 234, 194, 118, and 72 bp.

All 16 ‘*Ca*. P. solani’-related strains yielded *Vmp*1 gene amplicons of around ~1.4 kbp length. All TYPH10F/R nested-PCR products showed identical lengths (Figure 6). However, the *Rsa*I digestion profiles of tomato and potato samples were found to be different, as seen in Figure 7. Regardless of the region from which it is isolated, all ‘Ca. P. solani’ isolates infecting tomatoes were found identical to each other in terms of partial *Vmp*1 gene fragments. Potato-infecting ‘*Ca*. P. solani’ isolates were found to be identical too; however, when the *Vmp*1 gene fragments infecting tomato and potato plants were compared, it was detected that 93.54% similarity according to the blast analysis. Representative sequences were selected and submitted to GenBank under the accession numbers ON885883, ON885884, and ON885885, respectively. 

A phylogenetic tree was built using *Vmp*1 gene sequences from the NCBI GenBank database to represent the various RFLP patterns. These sequences were differentiated from each other according to *Rsa*I-RFLP digestion profiles. *Rsa*I enzymatic digestion profiles with *Vmp*1 gene amplicons enabled the identification of two distinct RFLP types (Figure 7), according to different V types reported in the previous studies [21,29,39]. 

Different profiles have been obtained for *Vmp*1, similar to the *Sec*Y gene-based typing. While the *Rsa*I digestion profiles of the ‘*Ca*. P. solani’ *Vmp*1 gene fragments isolated from potatoes were found close to the V1 type, the digestion profile of *Rsa*I isolated from tomatoes was uniquely determined. The *Vmp*1 gene sequence variations varied in tomato and potato isolates. 

In this study, the phytoplasmas infecting the tomato and potato were also distinguished by their distinct *Vmp*1 profiles thanks to restriction digestion using *RsaI* enzymes (Figure 7). 

## 3. Discussion

The first report of stolbur disease was recorded in 1988 in Turkey [40]. Epidemic occurrences of tomato or potato stolbur disease were previously reported in different regions of Turkey [2,3,41,42]. As seen by previous studies, the association between ‘*Ca*. P. solani’-related strains and other phytoplasma species have already been noted in Turkey. However, very few studies report detailed characterization, such as MLST or multigene characterization of phytoplasma diseases in Turkey. By employing DNA sequences of the *16S rRNA*, *Tuf*, *Sec*Y, and *Vmp*1 genes of ‘*Ca*. P. solani’, this study emphasized that there could be different genetic variations infecting tomatoes and potatoes in Turkey. This study used a multiple gene sequence analysis to discriminate between closely similar ‘*Ca*. P. solani’ variations infecting crops belonging to the same family. The two provinces are about 200 km apart, and the altitude difference is 545 m. The genetic differences between the samples did not differ according to the region, and the most important difference was detected with the *Sec*Y and *Vmp*1 genes. It has been determined that this genetic difference between the pathogen originates from the cultivated plant from which it is isolated. The *16SrDNA* and *Tuf* partial gene sequences of positive samples from tomato and potato showed 100% identity with each other and >99% with reference strains. The conserved *16SrDNA* gene has been the main basis for the classification of phytoplasmas into ribosomal groups based on 16S and subgroups, as well as the naming of “*Candidatus* Phytoplasma” species. However, due to the high level of rRNA nucleotide sequence conservation across numerous phytoplasma lineages, identifications based only on the *16SrDNA* gene have some limitations. Therefore, different genes other than *16SrDNA* are generally used for further typing of phytoplasmas. The *Tuf* gene is more informative than *16SrDNA* for the epidemiological search of the ‘*Ca*. P. solani’ strains. The epidemiological informative and housekeeping gene-based data of ‘*Ca*. P. solani’ are combined in their genetic structure. Based on the nucleotide polymorphisms of the gene constitutive elongation factor Tu encoding (Tuf), ‘*Ca*. P. solani’ often belongs to one of two molecular types (tuf a, b) [38]. *Tuf*-b, one of the epidemiological types of ‘*Ca*. P. solani’ is mainly reported as *Convolvulus arvensis*-related (field bindweed). According to our findings, all ‘*Ca*. P. solani’-related isolates were found as *Tuf*-b type. The abundance of the *Tuf*-b-type-infecting tomato and potato plants indicates that the *Convolvulus arvensis*-related alternative host system is the primary component of ‘*Ca*. P. solani’-related strain ecology in the Adana and Kahramanmaraş provinces in Turkey agro-ecosystem. *Tuf*-b type and other strains of ‘*Ca*. P. solani’ was also reported in Iran and Azerbaijan, border neighbors with Turkey. If we look at vectorial transmission, *Hyalesthes obsoletus* Signoret (Homoptera: Cixiidae), a polyphagous insect vector that lives predominantly on bindweed (*Convolvulus arvensis* L.), and other hosts of ‘*Ca*. P. solani’ inside and/or outside agrosystems is responsible for the transmission of ‘*Ca*. P. solani’ to crop plants [38,43]. No vector or weed sampling was performed in our study; however, the presence of the *Hyalestes obsoletus* and *Convolculus arvensis* have been previously reported in Turkey [44,45]. According to data from this study, phytoplasmal diseases that may be transmitted by *Hyalestes obsoletus* may cause significant yield losses in these crops in the future. 

**Figure 8:**
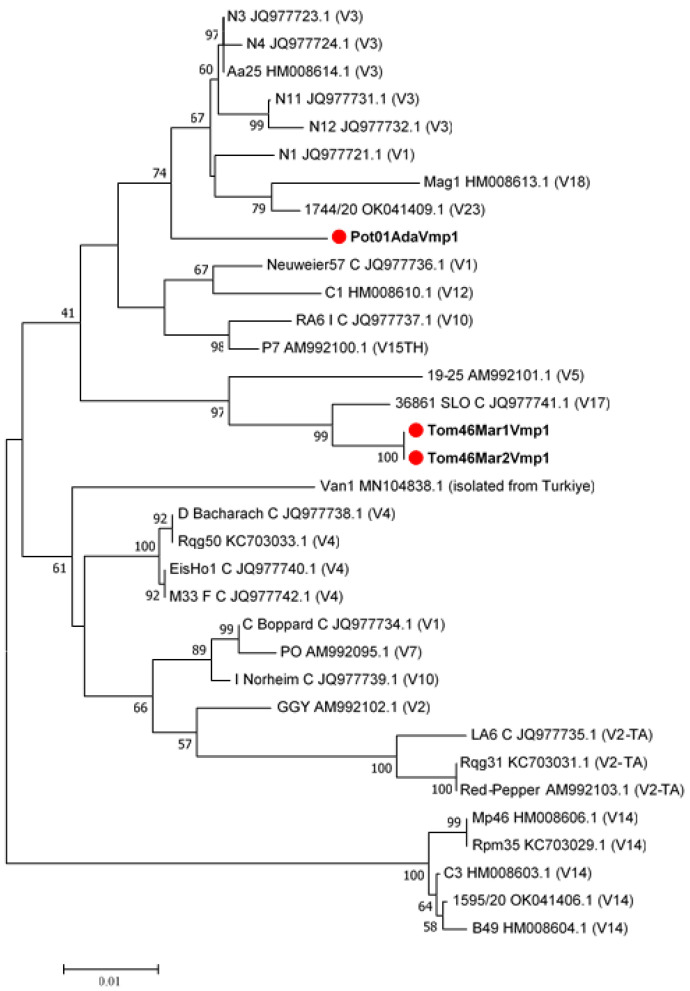
Unrooted phylogenetic tree constructed using ~1.4 kbp Vmp1 gene fragments of the ‘*Ca*. P. solani’ strains obtained in GenBank (Detailed information (host plant and origin country) about ‘*Candidatus* Phytoplasma solani’ strains used in the tree were provided in Supplementary Table S1). The phylogenetic analysis was performed with the neighbor-joining technique, and 1000 bootstrap replicates. The phytoplasma strain names, GenBank accession numbers, and related V types are given in the tree. The gene sequences obtained in this study are highlighted with red dots.

Recently, a study characterizing *Sec*Y and *Vmp*1 partial genes of the ‘*Ca*. P. solani’ strains was carried out in the Eastern Region of Turkey [46]. They found that the phytoplasma associated with tomato showed a highly phylogenetic affinity with the same sequences of the same agent from Serbia and France, respectively, based on *Vmp*1 and *Sec*Y gene sequences. The *Vmp*1 gene sequence was included in the phylogenetic analysis in this study (Figure 8). While all of the phytoplasma isolates infecting the tomato in our study formed a single clade, it was determined that they differed from the *Vmp*1 type detected in the previous study. We think this situation is probably due to the geographical region difference between Turkey. In our study, as seen in Figure 5, we identified two different *Sec*Y genotypes that were found to be genetically close to the SecY-4 type in central regions of Turkey that are closely related to those found in other countries infecting different insects or hosts.

According to the *Vmp*1 gene typing, by using *RsaI* digestion of TYPH10F/R products, we distinguished two new different profiles not reported in previous studies that have become prevalent in Turkish tomato and potato fields (Figure 7). ‘*Ca*. P. solani’-related strains isolated from potatoes were found to be similar to the V1 type. However, the strains isolated from tomatoes were not similar to any previously reported V variants of ‘*Ca*. P. solani’. Like all Mollicutes, phytoplasmas do not have a cell wall, and the role of membrane proteins in phytoplasma-host interactions has been emphasized by previous studies [36,47]. Furthermore, the newly discovered *Vmp*1 genotypes could raise the potential for another insect(s) or different host(s) to be involved in the disease dissemination of ‘*Ca*. P. solani’-related strains in Turkey. The Vmp1 gene provides information about the phytoplasma vector-pathogen-weed host relationships and the molecular epidemiology of the pathogen [36]. This gene is highly variable and interacts with hosts or vectors in complex ways. According to previous studies, it could be instructive and helpful for understanding the molecular epidemiology of ‘*Ca*. P. solani’ [21,25].

Further investigations that characterize ‘*Ca*. P. solani’-related strains infecting weed hosts (or other solanaceous plants) and planthoppers found in Turkish tomato and potato fields must confirm and better understand ‘*Ca*. P. solani’-related strains epidemiology. 

## 4. Material and Methods 

### 4.1. Sources of Nucleic Acids and Field Sampling

In August 2020 and 2021, 10 tomato and six potato fields occupying 10 ha intensive production areas in the central Anatolia region were surveyed to examine possible ‘*Ca*. P. solani’-associated symptoms. Leaf samples were collected from 30 tomato and 18 potato plants exhibiting typical phytoplasma symptoms in Kahramanmaraş and Adana provinces. At least one sample of an asymptomatic plant as a negative control was taken from each field. The samples were wrapped in paper, labeled, and put into plastic bags, then delivered to the laboratory in cold chain conditions on the same day. The samples were then cleaned of papers, sliced, and stored at −20 °C until use.

### 4.2. Isolation of Nucleic Acids and PCR Detection of Phytoplasmas

Total nucleic acid extractions were performed using fresh leaves (from midrib) of the samples to isolate and identify possible phytoplasmas. With a few minor adjustments, the extraction procedure was carried out utilizing the CTAB-based approach [48]. In short, midrib tissues (~1 g) were homogenized inside CTAB-buffer (2% *w*/*v* cetyl trimethyl ammonium bromide, 1.4 mol/L NaCl, 20 mmol/L EDTA, 100 mmol/L Tris HCl, 0.2% 2-β-mercaptoethanol and 2% polyvinylpyrrolidone-40, at pH 8.0) then it was left incubating at 65 °C for 25 min. The lysate was extracted by using a 24:1 (*v*/*v*) mixture of chloroform/isoamyl alcohol, then precipitated with isopropanol at 20 °C overnight. Centrifugation at 15,000× *g* was used to separate the precipitated nucleic acids, after which the pellet was cleaned with 70% ethanol, allowed to dry at room temperature, and then dissolved in 50 μL of sterile double-distilled water (SDDW). Before polymerase chain reaction (PCR) experiments, nucleic acids were measured and diluted with SDDW to a final concentration of 20 ng/μL. Stem parts of a cactus plant known to be infected with phytoplasma were used as a positive control in the DNA isolation.

Initial phytoplasma identification was made by nested-PCR analysis with phytoplasma universal primer pairs P1 [49]/Tint [50] amplifying a fragment of ~1.6 kbp from the *16SrRNA* gene and a part of the 16S–23S spacer region followed by R16F2n/R2 primer pairs [51] amplifying ~1.2 kbp products from the 16S rRNA. Pure DNA from maize plants known to be infected with the ‘*Ca*. P. solani’-related strain was used as a positive control in PCR-related analyses.

### 4.3. Molecular Characterization of the Strains Based on Tuf, VMP1, and SecY Genes

Characterizations of ‘*Ca*. P. solani’-related strains associated with tomato and potato were carried out based on the *Vmp*1 gene, which encodes a putative membrane protein, the *Tuf* gene, which encodes the translation elongation factor Tu, and the *Sec*Y gene, which encodes the translocase protein. All the phytoplasma-positive total nucleic acids amplified by the R16F2n/R2 primers were used as the template for amplification of *Vmp*1, *Tuf,* and *Sec*Y genes using StolH10F1/R1, fTuf1/rTuf1 and PosecF1/R1 primer pairs followed by nested-PCR with TYPH10F/R, fTufAY/rTufAY, and PosecF3/R3 primer pairs, respectively [36,52,53]. 

### 4.4. Sequencing and Phylogenetic Analyses 

ExoSAP-IT^®^ for PCR product clean-up (Thermo Fischer Scientific, Waltham, USA) was used to purify the obtained amplicons in accordance with the manufacturer’s instructions. The purified products were quantified utilizing a nanodrop spectrophotometer. Direct sequencing of all positive amplicons with high-quality bands in the agarose gel from several genes (*16SrDNA*, *Tuf*, *Sec*Y, and *Vmp*1) was performed by Letgen, Turkey, on both strands with the related primer pairs used for the amplification. Chromatograms were edited using the Chromas version 2.33 software (http://technelysium.com.au/wp/chromas/, accessed on 26 June 2022) and assembled using the MEGA 7 software [54] to obtain a consensus sequence, and it was used to cut off ~30–40 bp of the terminal end sequence. To find the nucleotide identities for the ‘*Ca*. P. solani’ strains, the NCBI GeneBank database (http://www.ncbi.nlm.nih.gov, accessed on 26 June 2022) was searched using all of the consensus sequences for each gene as the query sequences (http://blast.ncbi.nlm.nih.gov/Blast.cgi, accessed on 26 June 2022).

The nucleotide identities for the ‘*Ca*. P. solani’ strains are available in GeneBank NCBI (http://www.ncbi.nlm.nih.gov, accessed on 26 June 2022), and all of the consensus sequences for each gene were used as the query sequences in the BLAST searches (http://blast.ncbi.nlm.nih.gov/Blast.cgi, accessed on 26 June 2022). Multiple alignments were carried out using ClustalW, and nucleotide sequences were collected in FASTA format [55].

Phylogenetic trees based on distance analysis were produced by Mega 7 [54]. As indicated next to the tree branches, the proportion of replicate trees in which the related strains grouped in the bootstrap test (1000 repetitions) was calculated. The scaled phylogenetic trees had branch lengths that corresponded to the evolutionary distances that were utilized to build the trees. As an outgroup for the 16S rRNA tree root, the sequence from *Acholeplasma laidlawii* (GenBank accession number M23932) was employed. In order to rebuild the evolutionary connections according to the *16SrDNA*, *Tuf*, *Vmp*1, and *Sec*Y genes, various nucleotide sequences were retrieved from the GenBank database and used together with the nucleotide sequences generated in this work.

Different nucleotide sequences were downloaded from the GenBank database and used together with the nucleotide sequences generated in this study to reconstruct the phylogenetic relationships according to *16SrDNA*, *Tuf*, *Vmp*1, and *Sec*Y genes. 

### 4.5. In Silico RFLP Analyses

Since all amplicons with positive results were sequenced, a wet restriction fragment length polymorphism (RFLP) study was not preferred; instead, in silico RFLP analysis was performed with these sequences via iPhyClassifier [56] based on the *16S rRNA* gene sequences with 17 different restriction endonuclease enzymes to determine the structural diversity [57]. The pDRAW32 software v. 1.1.146 (AcaClone software, http://www.acaclone.com (accessed on 26 June 2022)) was employed for virtual digestion of the *tuf* and *vmp1* sequences with the *Hpa*II and *Rsa*I endonucleases, respectively.

## Data Availability

Sequence data reported in this study can be found at NCBI.

## Supplementary Materials

The following is available online at https://www.mdpi.com/article/10.3390/pathogens11091031/s1, Table S1: Candidatus Phytoplasma solani’ genome project and genetic diversity in the Euro-Mediterranean basin.
